# New Insights into the Role of Epithelial–Mesenchymal Transition during Aging

**DOI:** 10.3390/ijms20040891

**Published:** 2019-02-19

**Authors:** Francisco Santos, Cristiana Moreira, Sandrina Nóbrega-Pereira, Bruno Bernardes de Jesus

**Affiliations:** 1 Department of Medical Sciences and Institute of Biomedicine—iBiMED, University of Aveiro, 3810-193 Aveiro, Portugal; franciscojfsantos@ua.pt (F.S.); cristi.moreira@ua.pt (C.M.); sandrina.pereira@medicina.ulisboa.pt (S.N.-P.); 2 Instituto de Medicina Molecular João Lobo Antunes, Faculdade de Medicina, Universidade de Lisboa, 1649-028 Lisboa, Portugal

**Keywords:** epithelial–mesenchymal transition, EMT, aging, fibrosis, cellular reprogramming

## Abstract

Epithelial–mesenchymal transition (EMT) is a cellular process by which differentiated epithelial cells undergo a phenotypic conversion to a mesenchymal nature. The EMT has been increasingly recognized as an essential process for tissue fibrogenesis during disease and normal aging. Higher levels of EMT proteins in aged tissues support the involvement of EMT as a possible cause and/or consequence of the aging process. Here, we will highlight the existing understanding of EMT supporting the phenotypical alterations that occur during normal aging or pathogenesis, covering the impact of EMT deregulation in tissue homeostasis and stem cell function.

## 1. Introduction

Epithelial–mesenchymal transition (EMT) is a biological process that allows static epithelial cells to transdifferentiate into cells with a mesenchymal phenotype [1]. Important features that characterize epithelial cells include cell–cell adhesion and an apical-basal polarity, which are established through the arrangement of tight junctions, adherens junctions, desmosomes, and gap junctions [2]. These cells are placed on a basement membrane and form one or more layers that act as a barrier that delineates tissues and organs [3,4].

Cells within certain epithelia are able to move back and forth between epithelial and mesenchymal states through EMT and its reverse, the mesenchymal–epithelial transition (MET) [5]. The conversion of epithelial cells to mesenchymal cells is particularly important during embryogenesis, mainly in the establishment of body plan and organogenesis. In later adult stages, the processes underlying EMT can be reactivated for wound healing and tissue regeneration [6]. Nevertheless, pathological activation of EMT can adversely cause organ fibrosis and is also implicated in cancer, contributing to tumor progression and metastasis [2,6]. Indeed, cancer and aging are two common traits of the aging process [7]. A better understanding of how aging results in tissue dysfunction and/or cancer is an important question nowadays and may result in strategies circumventing age-related pathologies [8].

During EMT, epithelial cells undergo a significant phenotypic alteration that starts with the loss of cell–cell junction proteins, such as E-cadherin, desmoplakin, occludin and connexin, which, consequently, leads to the loss of the apical-basal polarity. These alterations are accompanied by the upregulation of mesenchymal-type N-cadherin, and the accumulation of fibronectin in the extracellular matrix (ECM). Furthermore, the cytoskeleton goes through a significant rearrangement, which involves the replacement of cytokeratin by vimentin, resulting in changes in cell shape, going from a cuboidal to a spindle form [1,2,5,9,10,11]. Newly formed mesenchymal cells acquire a front-rear polarity and favor cell-ECM rather than cell–cell adhesions. These cells exhibit motility and, in some cases, gain the ability to degrade ECM proteins by matrix metalloproteases (MMPs), giving rise to an invasive behavior [2,4,6]. Recent data suggest that, because EMT is a transitional process, several intermediate states can be observed, and, in different contexts, many of these intermediate phenotypes can be considered the final states. Partial EMT states have been observed in embryonic development, cancer and fibrosis (reviewed in reference [12]).

Epithelial–mesenchymal transition is triggered by many signaling pathways and transcription factors, both in physiological and pathological settings [13]. Transforming growth factor-β (TGF-β) is considered the most potent activator of EMT, leading to the activation of signaling pathways that culminate in the expression of genes that encode EMT transcription factors (EMT-TFs). The three main families of EMT-TFs with essential roles in EMT include SNAI (Snail and Slug), TWIST (TWIST1 and TWIST2), and ZEB (ZEB1 and ZEB2) [4,14,15,16]. These transcription factors repress E-cadherin expression, therefore contributing to the disassemble of cell–cell junctions and leading to the induction of EMT [17]. Furthermore, Snail and ZEB repress the expression of certain genes that have an important role in cell polarity, namely *CRB1* (which encodes the Crumbs protein), whose inactivation enhances signaling by TGF-β, strengthening the EMT process [18,19,20]. Nevertheless, other growth factors (e.g., insulin-like growth factor (IGF), fibroblast growth factor (FGF), and epidermal growth factor (EGF)) and signaling pathways (e.g., Hedgehog and Wnt) can also trigger EMT by the expression of the aforementioned transcription factors [13,14].

Non-coding RNAs, such as microRNAs (miRNAs) and long non-coding RNAs (lncRNAs) [21], also play an important role in EMT by regulating the expression of EMT-TFs [14]. Several miRNAs reduce the expression of EMT-TFs; for example, members of the miR-200 family target ZEB factors, preventing the downregulation of E-cadherin and the initiation of EMT, contributing to the maintenance of the epithelial phenotype [22]. Tumor suppressor p53 also plays a role in negatively regulating EMT by inducing miRNAs that target EMT-TFs. For instance, p53 inhibits Snail and ZEB1 by inducing miR-34 and miR-200c, respectively [23,24]. Furthermore, p53 upregulates MDM2 and forms a complex of p53-MDM2-Slug to promote degradation of Slug, leading to the increased expression of E-cadherin [25]. In contrast, miR-544a and miR-21 act by targeting epithelial differentiation markers, thus promoting EMT [26,27]. Similarly, lncRNAs ZEB1 antisense 1 (ZEB1-AS1) and ZEB2 natural antisense transcript (ZEB2-NAT) promote the expression of ZEB1 and ZEB2, respectively, leading to increased metastasis and poor prognosis in numerous types of cancer [28].

Several reports have shown that sirtuins (SIRT), a family of class III histone deacetylases, may also play a role in EMT, acting as both enhancers and repressors of this process [29,30,31]. Sirtuin 1 is involved in aging [32,33,34], and in numerous types of cancer, such as prostate cancer, where ZEB1 recruits SIRT1 to the E-cadherin promoter. Sirtuin 1 deacetylates histone H3 and reduces binding of RNA polymerase II to the promoter, thus suppressing E-cadherin expression [35]. It has been suggested that SIRT1 plays a role in recruiting SIRT7 to the E-cadherin promoter, and that this interaction is responsible for inducing EMT [36]. Other SIRTs involved in EMT are SIRT2 and SIRT4. Overexpression of SIRT2 leads to an increased expression of Slug, resulting in a stronger repression of E-cadherin [37]. Sirtuin 4, on the other hand, is associated with an upregulation of E-cadherin and a reduced expression of vimentin via inhibition of glutamate dehydrogenase, thus blocking glutamine metabolism [38].

Due to the involvement of EMT in different pathways, it was proposed to classify EMT into three subtypes, based on the biological context that they occur in [6,39]. Type 1 EMT plays a role during embryogenesis and organ development and gives rise to cells with the potential to undergo the MET process, thus generating epithelial cells. Type 1 EMT neither causes fibrosis nor induces an invasive phenotype [6]. Type 2 EMT acts during organ fibrosis, wound healing, and regeneration, usually occurring after tissue injuries. Examples of organ fibrosis through type 2 EMT occur in the liver, lung, and kidney, which are explored further in this review. Several markers have been employed to distinguish epithelial cells undergoing EMT, for instance during inflammation. Amongst them are type 1 collagen, α-SMA (α-smooth muscle actin), vimentin, desmin, discoidin domain receptor 2 or FSP1 (fibroblast-specfic protein 1) [40,41,42,43,44,45]. Type 2 EMT can sustain tissue fibrosis until ongoing inflammation. Lastly, type 3 EMT occurs during cancer progression and this transition is involved in the acquisition of the potential to migrate and colonize distant organs [6,46]. During neoplastic progression, cancer cells acquire genetic and epigenetic marks affecting oncogenes and tumor suppressors, eventually resulting in the activation of type 3 EMT programs, giving them potential to invade and metastasize. Importantly, type 3 EMT is not equal for all cancer cells. Some cells may retain epithelial markers, while others may have both epithelial and mesenchymal markers or be fully mesenchymal. It is, however, unclear which signals give cancer cells through the EMT process [47]. Despite the differences between EMT subtypes, a common network may underline similarities between the processes, which could guide to mutual activators or repressors [47].

Our previous observations on the increased expression of Zeb2 during biological aging and their involvement as a barrier during cellular reprogramming of aged cells [28] motivated us to address whether EMT may be involved in the progression of age-related pathologies, and whether EMT is limiting the acquisition of stem cell properties restraining the reprogramming of aged cells.

## 2. EMT Balance during Biological Aging

**Figure 1:**
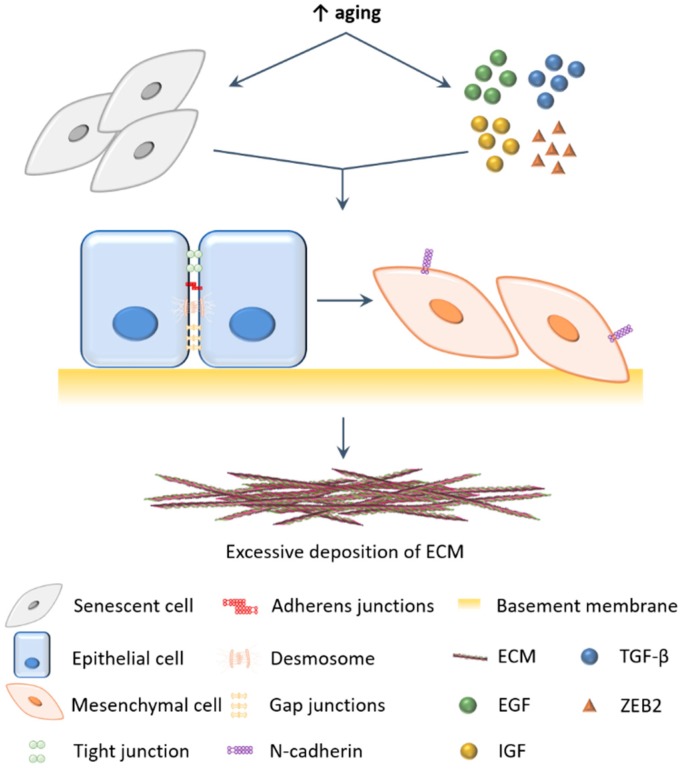
Role of epithelial-mesenchymal transition (EMT) in aging. Pro-EMT signals (eg. transforming growth factor-β (TGF-β), epidermal growth factor (EGF), insulin-like growth factor (IGF) and transcription factor ZEB2) and senescent fibroblasts with a senescent-associated secretory phenotype (SASP) accumulate in aged tissues, which are responsible for the induction of EMT. Epithelial cells lose their cell–cell junctions and apical-basal polarity. Cells begin to express N-cadherin, acquire front-rear polarity and gain motility, thus transdifferentiating into mesenchymal cells, such as fibroblasts. Fibroblasts produce extracellular matrix (ECM) components and are the main cellular mediators of fibrosis. When these cells accumulate (e.g., due to EMT), excessive fibrotic tissue is formed, affecting the function of vital organs. This figure was produced using Servier Medical Art.

Aging is a complex and multifactorial process characterized by the functional decline of cells, tissues, and organs, and is accompanied by an increased risk of the development of age-related diseases [7,8,48]. Amongst other characteristics, advanced age is known to contribute to pathological fibrosis and has been recognized as a risk factor for fibrotic disorders [49,50,51,52]. Tissue repair and regeneration is an essential process for maintaining the integrity and survival of organisms; however, some of the mechanisms involved become less reliable with aging, resulting in a decreased repairing capacity and progressive loss of tissue structure and function [53,54]. Fibroblasts, the major cellular mediators of fibrosis, are responsible for the deposition of ECM components (Figure 1) and, when these cells accumulate, an excess of fibrotic tissue is formed, compromising the function of vital organs [44,55,56]. Some fibroblasts have been proven to derive from epithelial cells that have undergone EMT, suggesting that this process has an important role in tissue fibrosis [45,57]. As example, EMT has been considered a key process that contributes to kidney fibrosis and the decline of renal function [58]. E-cadherin and α-SMA, which is a specific marker for mesenchymal fibroblasts, were found, respectively, in lower and higher levels in old rats, suggesting that kidney epithelial cells experience EMT to originate fibroblasts [59]. Likewise, in the heart (see Table 1), a similar EMT process, named endothelial-to-mesenchymal transition (EndMT), is responsible for the emergence of fibroblasts that originate from endothelial cells. The excessive deposition of ECM leads to cardiac fibrosis, which is common in patients with advanced cardiac failure [45]. Similarly, fibrosis is characteristic of cardiovascular pathology in accelerated aging syndromes such as Hutchinson–Gilford progeria syndrome (HGPS), comparable to the cardiovascular pathologies observed in geriatric patients [60]. Additionally, differential TGF-β signaling has been found to be altered in older cells, closely resembling the profiles in progeroid cells of patients with HGPS [61]. Furthermore, idiopathic pulmonary fibrosis (IPF), characterized by the loss of respiratory function due to the excessive deposition of ECM, exhibits an abnormal Wnt/β-catenin signaling pathway, which can induce EMT [62]. In normal adult lungs, the expression of β-catenin is restricted to cell membranes in endothelial and epithelial cells. However, in IPF patients, β-catenin is present in the nucleus, suggesting the involvement of the Wnt/β-catenin pathway in EMT [62]. Additionally, IGF-II seems to be responsible for inducing EMT and may contribute to the relocation of β-catenin to the nucleus during the process [63]. Similarly, impairment of the properties of the blood–brain barrier (BBB) is a key event during several diseases, including multiple sclerosis [64], or human aging [65]. Under these conditions, EndMT may have a potential role during BBB dysfunction in neurological disorders [64] and probably normal human aging. Indeed, studies in the living human brain and post-mortem tissue demonstrated the collapse of the BBB in Alzheimer’s disease (AD) and other neurodegenerative disorders [66]. Alzheimer’s disease is a complex neurodegenerative disorder characterized by specific biomarkers, including the disease-specific transcriptional biomarker Alz, which appears later in pathogenesis and is enriched in genes associated with EMT [67].

Senescent cells may also play an important role in inducing EMT. These cells are characterized by a permanent cell cycle arrest and increase with age in mammalian tissues [69]. Furthermore, senescent cells may have harmful effects on tissue microenvironment and may contribute to age-related diseases, having been found at sites of some of these diseases, such as osteoarthritis and atherosclerosis [70,71]. Senescent cells, including senescent fibroblasts, acquire a senescent-associated secretory phenotype (SASP), which is characterized by the increased secretion of certain cytokines, chemokines, and growth factors. Senescent-associated secretory phenotype derived from senescent fibroblasts has the ability to induce EMT in neighboring epithelial cells and has been shown to contribute to EMT in non-aggressive human breast cancer cell lines [70,71]. These cells, treated with conditioned medium from senescent cells, showed a decreased expression of E-cadherin, cell membrane-associated β-catenin and cytokeratin, and an increased expression of vimentin, which are all hallmarks of EMT. Interestingly, blocking antibodies against interleukins IL-6 and IL-8 seems to decrease the invasion stimulated by the conditioned medium of senescent cells. Furthermore, adding IL-6 and IL-8 to the conditioned medium of pre-senescent cells seems to promote invasion [70]. In the prostate, a greater amount of hepatocyte growth factor (HGF) was found to be present in the conditioned medium of senescent fibroblasts in comparison to that of pre-senescent fibroblasts. Hepatocyte growth factor is associated with the disintegration of cell–cell junctions, the disruption of epithelial cell morphogenesis, and the stimulation of migration and invasion, thus promoting EMT in surrounding epithelia [72,73,74]. An age-related change in the expression levels of some growth factors, namely TGF-β, EGF, and IGFs, is likely to contribute to the increase of EMT with age and play an important role in progressive fibrosis [53]. Moreover, the expression of HGF has been found to be higher in skin fibroblasts from old individuals and in response to IGFs that increase with aging [75,76]. p53 may also play a role in targeting the SASP. Coppé and colleagues found that p53 prevents the SASP, meaning that cells that lacked p53 secreted higher levels of many of the SASP components [70], further suggesting a role of p53 in preventing EMT. However, an age-associated decline in p53 activity has been observed [77]. In this study, young and old mice were treated with ionizing radiation and the tissues were examined for the p53 protein. They found that tissues from old mice showed significantly lower levels of p53 compared to their young counterparts [77]. Furthermore, a subsequent study revealed that the deletion of p53 in mice at 12 months resulted in a significantly faster tumor incidence than mice at three months, emphasizing the fact that p53 function becomes progressively more important in preventing cancer as the organism ages [78]. Given the decline of p53 levels with aging, it is reasonable to suggest that the prevention of SASP by p53 becomes less effective as individuals get older, meaning that SASP-specific components would accumulate and contribute to the induction of EMT and, therefore, play a role in tissue fibrosis.

### Is EMT Truly Involved in Liver Fibrosis?

Several studies have been trying to respond to whether EMT is behind liver fibrosis. Under experimental conditions, two major types of liver cells can undergo EMT: hepatocytes and cholangiocytes [79]. Incubation of hepatocytes with TGF-β leads to EMT, with cells losing epithelial markers and acquiring mesenchymal markers (such as type I collagen and vimentin). Studies in mice are inconclusive as to whether EMT may have a role in liver fibrosis. In mice, EMT does not contribute to liver fibrosis through the observation that epithelial cells cannot be converted into mesenchymal cells [80,81]. Similarly, EMT in cholangiocytes was recently assessed with lineage-tracing methodology [82]. Following labeling of cholangiocytes, they concluded that EMT of cholangiocytes does not participate in the biogenesis of liver fibrosis in mice.

On the other hand, evidence supporting EMT during liver fibrosis was confirmed in humans and in vitro. Expression of several mesenchymal markers has been observed in parenchymal cells of patients with liver diseases [83,84,85]. Namely, EMT markers were found in patients with several liver conditions, such as primary biliary cholangitis, non-alcoholic steatohepatitis [83,86], biliary atresia and primary sclerosing cholangitis [87]. These results support the involvement of EMT in chronic liver disease, particularly demonstrating the expression of mesenchymal markers in parenchymal cells. Further evidence supporting EMT during liver fibrosis comes from in vitro experiments showing that rat hepatocytes treated with TGF-β undergo EMT (assessed through the detection of high levels of vimentin, Snail and other mesenchymal markers) [88,89]. Additionally, stimulation of murine primary hepatocytes with TGF-β leads to the acquisition of mesenchymal markers and loss of epithelial characteristics [80,90,91]. TGF-β induces EMT through the regulation of transcriptional factors such as Snail, which is involved in the regulation of EMT [92]. Snail, alone, is able to induce EMT in adult hepatocytes. As with hepatocytes, rat cholangiocytes isolated after bile duct ligation show reduced expression of epithelial markers [86]. Additionally, administration of TGF-β induces mesenchymal characteristics in cultured primary human cholangiocytes [93]. TGF-β has been recognized as a critical factor leading to collagen deposition during fibrogenesis. In vitro, it was previously reported that TGF-β1 induces EMT in hepatocytes through the activation of Snail and Smad2/3 [91]. Similarly, in mature hepatocytes, claudin-1 was repressed by TGF-β, leading to EMT [92]. On the other hand, blocking TGF-β signaling by Smad7 in hepatocytes strongly impacts the fibrogenic response. Curcumin, schizandrin, and propolis were shown to inhibit EMT through interference with TGF-β signaling [94,95]. The TGF-β network is a major inducer of EMT. Despite contradictory results on their involvement on liver fibrosis [96,97], further studies are needed to elucidate whether TGF-β participates in fibrotic liver pathobiology. In addition to TGF-β, several pathways have been associated with EMT during liver fibrosis (reviewed in reference [47]). One well-documented pathway is the Hedgehog signaling (Hh) pathway, which is crucial in tissue remodeling and organogenesis [47,98]. Hh activation seems to be implicated in the formation of fibrotic tissue through EMT [84,86,99,100,101,102]. As an example, during non-alcoholic fatty liver disease (NAFLD), epithelial genes are suppressed by Sonic Hh, and activation of the Hh pathway in mouse models of NAFLD leads to EMT [84].

Currently, the mechanism supporting or not the involvement of EMT during liver fibrosis is not fully elucidated. A detailed comprehension of EMT and its exact role during organ fibrosis upsurges as a request for designing anti-fibrotic targeted therapeutics.

## 3. EMT in Acquired Stem Cell Properties during Aging

The ability of stem cells to maintain pluripotency is one of their most important characteristics. However, with aging, the regenerative potential of stem cells declines [103,104,105,106]. Aging of stem cells is the result of several intrinsic and extrinsic factors, such as the accumulation of cellular damage and toxic metabolites, loss of proteostasis, mitochondrial dysfunction, and telomere shortening [7,107,108,109,110,111]. Together, these factors can lead to senescence, cell death, and stem cell exhaustion. Like differentiated cells, stem cells are also unprotected from stimuli that promote aging [112]. For instance, the decrease in cell cycle activity of hematopoietic stem cells (HSCs) was demonstrated by studies in aged mice [113,114] and there is evidence correlating the increased FGF signaling to the loss of quiescence in the aged muscle stem cell niche leading to stem cell depletion and diminished self-renewing capacity [115]. We have previously demonstrated that Zeb2 was early activated in mouse embryonic stem (ES) cells in the absence of pluripotency conditions. Both EMT and MET are key processes during cellular transdifferentiation [15]. During development, EMT is responsible for the formation of the neural crest delamination, heart valve differentiation and lung organogenesis [2]. Conversely, during adulthood, connective tissue growth factor (CTGF) has been shown to produce stem-like properties in cancer cells through MET, exhibiting characteristics of cancer stem cells [116]. Additionally, several EMT genes have been correlated with the presence of stem-like characteristics in cancer cells. For instance, induction of EMT in immortalized human mammary epithelial cells (HMLEs) resulted in the acquisition of mesenchymal traits and in the expression of stem cell markers. Epithelial cells that undergo EMT not only gain mesenchymal traits, but also acquire features that are characteristic of stem cells [117]. HMLEs, after being induced to go through EMT, by the expression of Snail or TWIST, developed a CD44^high^CD24^low^ expression pattern, which is distinctive of stem cells, including normal mammary epithelial stem cells and human breast cancer stem cells (CSCs) [117]. This provides a link between EMT and features associated with CSCs, including self-renewal and the ability to produce differentiated non-stem cells [118]. These CD44^high^CD24^low^ cells were also characterized by markers associated with EMT, namely a decreased expression of E-cadherin and an increase of the expression of fibronectin and vimentin, and exhibited increased tumorigenic properties [117]. For a concise review on the role of EMT-TFs in tumorigenesis see reference [119].

## 4. Role of EMT during the Reprogramming of Aged Cells

In 2006 and 2007, Yamanaka and colleagues described how the expression of four transcription factors in, respectively, mice skin and adult human fibroblasts rejuvenate their properties to a “stem-like” condition named induced pluripotent stem cells (iPSCs) [120,121]. Induced pluripotent stem cell reprogramming was a major advance for the study of tissue plasticity, in vitro tissue generation, and tissue regeneration strategies. However, several age-related processes have a negative impact on the properties of different cells and tissues, turning them refractory to the reprogramming process. As previously considered, the efficiency of reprogramming of adult cells into iPSCs decreases with aging [122,123]. For instance, the increased expression of the tumor suppressors p16INK4a and p53 during aging are barriers to the reprogramming of somatic cells into iPSCs, and their absence facilitates the reprogramming of aged cells.

During the reprogramming of somatic cells, cells of mesenchymal origin, such as fibroblasts, need to undergo MET to generate intermediate cells with epithelial features (and ultimately iPSC) under the influence of the Yamanaka reprogramming factors (Oct4, Sox2, c-Myc and Klf4) [14,48,124]. Oct4 and Sox2 suppress EMT-inducing transcription factor Snail, c-Myc downregulates TGF-β1 and its receptors, and Klf4 induces the expression of E-cadherin. These factors, together with bone morphogenic protein 7 (BMP7), are responsible for repressing TGF-β signaling, thus preventing the EMT process and promoting an epithelial phenotype [15,125]. Cells must acquire an epithelial phenotype before they fully reprogram into iPSCs, probably due to the epithelial nature of pluripotent embryonic stem cells from the inner mass of the blastocyst [73,126]. It has been previously shown that reprogramming mouse embryonic fibroblasts and human adult fibroblasts into iPSCs requires a sequential EMT–MET process, during which cells express EMT-TFs and exhibit an enhanced motility before acquiring an epithelial phenotype [125]. In line with the involvement of MET during reprogramming, blocking the action of certain growth factors (e.g., IGF-I) with chemical inhibitors has shown to enable MET and, therefore, improve cell reprogramming [127]. Also, fibroblasts from old mice have been shown to express higher levels of ZEB2 compared to fibroblasts from young mice, contributing to the inefficient reprogramming of old fibroblasts [28]. The DNA binding domains of ZEB2 (and ZEB1) are composed of two zinc finger clusters located towards the N and C-terminal ends. These zinc-finger clusters bind to a CACCTG DNA motif called E-box. In addition to being important for DNA binding, the Zn-finger domains also mediate physical interactions with other transcription factors. These domains of ZEB2 interact, for instance, with Pc2 [128,129]. ZEB2 was initially described as a transcriptional repressor [130], in particular as an EMT-inducer, by repressing E-cadherin and other epithelial genes. ZEB factors repress transcription through competing and displacing transcriptional activators from their binding sequences in the DNA [131,132]. Several reports have shown that post-translational modifications of ZEB factors also contribute to the activator or repressor switch [133,134]. In human cells, ZEB2-NAT, which is a lncRNA antisense of ZEB2, regulates the latter’s expression and increases with aging, further contributing to an inefficient reprogramming. LncRNAs were shown to play important regulatory roles in modulating transcriptionally networks and altering transcriptionally and post-transcriptionally the coding transcriptome [21,135,136]. Downregulation of ZEB2-NAT leads to a decreased expression of ZEB2, therefore improving reprogramming [28]. Similar to Zeb2/Zeb2-NAT, BMP can induce the expression of the miR-200 and miR-205 families of miRNAs, suppressing ZEB1 and ZEB2 expression [137,138]. Furthermore, miR-302 and miR-372 contribute to the downregulation of TGF-β receptor type II, increasing the efficiency of reprogramming [139].

It is worth mentioning that several lncRNAs have been shown to regulate EMT transcription factors. Examples are the lncRNA LEIGC, regulating EMT in gastric cancer, HOTAIR, or Zeb1-AS1 [124,140,141]. Several lncRNAs have been shown to target directly the EMT markers, through mechanisms not fully described. LncRNAs DREH and AOC4P, for instance, regulate vimentin expression during tumor metastasis or enhances vimentin degradation during hepatocellular carcinoma, respectively, leading to tumor suppressive effects [142,143,144]. In summary, EMT is regulated by several pathways, many of which regulated through lncRNAs. Although much of the knowledge linking EMT and lncRNAs was assessed from the biology of cancer, EMT regulation by lncRNAs may prove fundamental for other molecular processes.

## 5. Future Directions

Here, we discussed the possible involvement of EMT during the aging process. Whether attenuation of the EMT pathway may act positively on age-related pathologies is still unknown. Metformin, an extensively used anti-diabetic drug that has been shown to protect against several age-related pathologies [145,146,147,148], directly binds to and inhibits TGF-β1, suppressing TGF-β1 receptor dimerization and downstream signal transduction [149]. Indeed, it was previously observed that TGF-β1-deficient mice survived longer and exhibited less myocardial fibrosis compared to age-matched controls [150].

In a similar way, caloric restriction, which is characterized by a decreased food intake, avoiding malnutrition, delays the aging process in several species. Interestingly, caloric restriction in humans shows significantly lower TGF-β1 levels compared with the Western diet group (29.4 ± 6.9 ng/mL to 35.4 ± 7.1 ng/mL, respectively) [151]. These results guide to the possible targeting of EMT as an anti-aging strategy.

## Figures and Tables

**Table 1:** Expression of epithelial and mesenchymal markers in fibrotic organs.

Organ	Characteristics	Reference
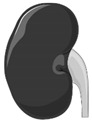	↓ Expression of E-cadherin ↑ Expression of α-smooth muscle actin (SMA) ↑ Expression of Zeb1	[59]
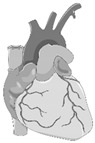	Fibroblasts marked with β-gal and FSP-1 ↑ Expression of TGF-β mRNA ↑ Of phosphorylated Smad2/3 in the nucleus	[45]
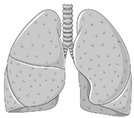	Nuclear β-catenin ↓ Expression of E-cadherin ↑ Expression of α-SMA, fibronectin and vimentin	[62,68]
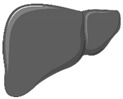	Fibroblasts marked with β-gal and FSP-1 ↓ Expression of E-cadherin	[45]
